# A randomised trial comparing 6-monthly adjuvant zoledronate with a single one-time dose in patients with early breast cancer

**DOI:** 10.1007/s10549-024-07443-2

**Published:** 2024-07-31

**Authors:** Arif Ali Awan, Carol Stober, Gregory R. Pond, Igor Machado, Lucas Clemons, Henry Conter, Demetrios Simos, Sukhbinder Dhesy-Thind, Mihaela Mates, Vikaash Kumar, John Hilton, Marie-France Savard, Dean Fergusson, Lisa Vandermeer, Mark Clemons, the REaCT Investigators

**Affiliations:** 1 Division of Medical Oncology, Department of Medicine, The Ottawa Hospital and University of Ottawa, Ottawa, Canada; 2 Cancer Therapeutics Program, Ottawa Hospital Research Institute, Ottawa, ON Canada; 3 Escarpment Cancer Research Institute and McMaster University, Hamilton, Canada; 4 William Osler Cancer Centre and Department of Oncology, Brampton, Canada; 5 Stronach Regional Cancer Center, Newmarket, Canada; 6 Department of Oncology, Cancer Centre of Southeastern Ontario and Queen’s University, Kingston, Canada; 7 Shakir Rehmatullah Cancer Clinic, Markham Stouffville Hospital, Markham, Canada; 8 Clinical Epidemiology, Ottawa Hospital Research Institute and Departments of Medicine, Surgery, and the School of Epidemiology and Public Health, University of Ottawa, Ottawa, Canada; 9 Division of Medical Oncology, The Ottawa Hospital Cancer Centre, 501 Smyth Road, Ottawa, K1H 8L6 Canada

**Keywords:** Breast cancer, Adjuvant, Bisphosphonates, Zoledronate

## Abstract

**Purpose:**

While adjuvant bisphosphonate use in early breast cancer (EBC) is associated with improvements in breast cancer-specific outcomes, questions remain around optimal bisphosphonate type, dose and scheduling. We evaluated a single zoledronate infusion in a prospective randomised trial.

**Methods:**

Postmenopausal patients with EBC were randomised to receive a single infusion of zoledronate (4 mg IV) or 6-monthly treatment for 3 years. Outcomes measured were; Quality of Life (QoL; EQ-5D-5L), bisphosphonate-related toxicities, including acute phase reactions (APRs), recurrence-free survival (RFS), bone metastasis-free survival (BMFS) and overall survival (OS).

**Results:**

211 patients were randomized to either a single infusion (n = 107) or six-monthly treatment (n = 104). After 3 years of follow up there were no significant differences between the arms for QoL and most toxicity endpoints. APRs following zoledronate occurred in 81% (171/211) of patients (77.6% in single infusion arm and 84.6% in the 6-monthly group). While the frequency of APRs decreased over 3 years in the 6-monthly arm, they still remain common. Of 34/104 (32.7%) patients who discontinued zoledronate early in the 6-monthly treatment group, the most common reason was APRs (16/34, 47%). At the 3 year follow up, there were no differences between arms for RFS, BMFS or OS.

**Conclusion:**

A single infusion of zoledronate was associated with increased patient convenience, less toxicity, and lower rates of treatment discontinuation. Despite the common clinical impression that APRs decrease with time, this was not observed when patients were specifically questioned. While the study is not powered for non-inferiority, longer-term follow-up for confirmation of RFS and OS rates is ongoing.

**Supplementary Information:**

The online version contains supplementary material available at 10.1007/s10549-024-07443-2.

## Introduction

Despite randomised trials, meta-analyses and evidence-based guidelines [1–6], the uptake of adjuvant bisphosphonates remains variable [7–9]. Sources of this variability include questions around optimal adjuvant bisphosphonate type, dose and scheduling in relation to drug efficacy, toxicity and patient convenience [7, 8]. With respect to efficacy, the Oxford meta-analysis of individual patient data was unable to identify any specific agent/regimen as being superior to others [6]. For toxicity, different agents and schedules are associated with their own toxicity profiles [10]. Given these challenges, the CCO-ASCO evidenced-based guideline stated, “further research comparing different bone-modifying agents, doses, dosing intervals, durations and better means to identify who would benefit the most from it, are required” [1].

The results of healthcare provider surveys suggest that the most commonly used strategy in clinical practice is 6-monthly zoledronate for 2 or 3 years [2, 7–9]. For this regimen, breast cancer-specific outcomes (iDFS and OS) are available from 1 trial with comparing zoledronate with no zoledronate [3, 11] and from 2 trials evaluating upfront versus delayed zoledronate for cancer treatment-associated bone loss [5, 12]. Toxicity outcomes for 6-monthly treatments are reported in 6 studies (Supplemental Materials Table S1) [3, 5, 11–15]. The most commonly reported toxicities include: APRs (30 to 57%), renal toxicity (1 to 4%) and osteonecrosis of jaw (ONJ) (0.1 to 0.2%) [3, 5, 11–15]. It is of note that trials in the osteoporosis [16–19], cancer therapy-associated bone loss [20, 21], metastatic bone disease [22–25], and adjuvant settings [26] have all confirmed the efficacy of less frequent use with less toxicity and greater patient convenience. Indeed, a single zoledronate infusion is associated with increased bone density for many years in both the osteopenia [18, 19] and cancer-associated bone loss [21] settings.

Given the positive findings from studies evaluating less frequent zoledronate in other settings, we performed a randomised control trial comparing a single infusion of adjuvant zoledronate with 6-monthly treatment. The primary endpoint of demonstrating the feasibility of physicians approaching, and patients entering, a study using less frequent dosing has been previously published [27]. In the current manuscript we present Quality of Life (QoL), bisphosphonate-associated toxicities, recurrence-free survival (RFS), bone metastasis-free survival (BMFS) and overall survival (OS) data after 3 years of follow-up.

## Methods

### Study design and participants

REaCT-ZOL is a pragmatic, multi-center, open-label study. Full details are described elsewhere [27]. Briefly, postmenopausal (either natural or treatment-induced) patients with histologically confirmed EBC deemed candidates for adjuvant zoledronate were eligible. Patients were required to start zoledronate within 3 months of completion of chemotherapy or within 3 months of starting endocrine therapy if not receiving chemotherapy. Patients had to have serum creatinine clearance > 30 mL/min and normal serum calcium as per their institution’s norm. Exclusion criteria included: metastatic disease or a history of ONJ. Eligible and consented patients were randomly assigned to either: a single infusion of zoledronate (4 mg) or 6-monthly infusions of zoledronate (4 mg) over 3 years for a total of 7 infusions (i.e. treatment on months 0, 6, 12, 18, 24, 30, 36).

### Quality of life

Quality of life was measured using the validated EQ-5D-5L questionnaire which evaluates five dimensions (mobility, self-care, usual activities, pain/discomfort and anxiety/depression) and each dimension has five response levels: no problems, slight problems, moderate problems, severe problems, unable to/extreme problems [28]. Responses are coded as single-digit numbers expressing the severity level selected in each dimension. The EQ-5D-5L also incorporates a visual analog scale for “How good is your health today?” This provides a measure of the patient’s perception of their overall health (0–100). Questionnaires were completed 5–7 days pre- and 5–14 days post-each infusion of zoledronate for those on the 6-monthly arm. Patients on the single infusion arm filled out EQ-5D-5L 5–7 days pre- and 5–14 days post-their infusion of zoledronate, then annually for 3 years. For those patients that stopped 6-monthly dosing early the EQ-5D-5L was completed every 6-months for 3 years.

### Acute phase reactions (APRs)

While the definition of APRs varies in the literature [3, 5, 12, 13, 15], we used the definition of Reid et al. [29]. In this scoring system APRs are reflected by five main groups of symptoms: fever symptoms (e.g. fever, chills, hot flushes), musculoskeletal symptoms (e.g. muscle pain, muscle stiffness, joint swelling), gastrointestinal (e.g. abdominal pain, reduced appetite, diarrhea, nausea and vomiting), eye symptoms (eye irritation, eye pain, eye swelling) and general symptoms (e.g. fatigue, dizziness, flu-like symptoms) [29]. As these symptoms have their peak incidence 1 day after the infusion with a median duration of 3 days [29] patients were contacted by telephone 5–14 days after each infusion and asked to if they had had any of these symptoms (yes/no) within 3 days of their zoledronate infusion. The occurrence and appropriate timing of any of these symptoms was considered to be an APR. During this telephone call patients were also asked if these symptoms had resulted in them needing to seek additional medical attention.

### Other bisphosphonate-associated toxicities

Serum creatinine clearance and calcium were measured prior to each zoledronate infusion. Bisphosphonate-associated toxicities such as impaired renal function resulting in either discontinuation of zoledronate or zoledronate dose adjustment, renal failure and hypocalcaemia were evaluated from patient blood results and patient/physician follow-up questionnaires that were performed every 6 months for 3 years. Questionnaires also collected information on new diagnoses of ONJ, atypical femur fractures, and atrial fibrillation. If ONJ was suspected, its diagnosis had to be confirmed by an oral surgeon.

### Bone health

Baseline FRAX® scores were calculated on all patients [30, 31]. FRAX incorporates both personal details and BMD results (if available). In the current study, BMD scores were incorporated if available within 3 years prior to randomization or 6 months afterwards. Baseline and 6-monthly physician/patient questionnaires were performed to collect information on a new diagnosis of osteoporosis and/or the occurrence of fragility fractures.

### Recurrence-free survival (RFS), bone metastasis-free survival (BMFS) and overall survival (OS)

RFS was defined as the time from randomization until the first occurrence of either ipsilateral invasive breast tumor recurrence, local/regional invasive breast cancer recurrence, distant recurrence, or death due to any cause [32]. BMFS was defined as the appearance of the cancer in the bone with radiological confirmation. OS was defined as the number of people alive, with or without signs of cancer.

### Compliance with study arm

Date of zoledronate administration was collected. Reasons for treatment discontinuation were collected for patients that chose to discontinue future treatments. If a patient chose to discontinue zoledronate earlier than planned in their study arm they were followed using patient/physician follow-up questionnaires every 6 months for 3 years.

### End points/outcomes

The primary endpoint was to demonstrate the feasibility of accruing patients to a study comparing a single zoledronate infusion to 6-monthly treatment (to provide supporting data for applying for funding for a larger definitive trial) [27]. The current manuscript presents secondary endpoints of QoL, bisphosphonate-associated toxicities, including acute phase reactions (APRs); and breast cancer-specific outcomes (RFS; BMFS; and OS).

### Statistical analysis

Descriptive statistics were used to summarize patient, treatment and outcome characteristics for all patients and by intervention arm separately. The Kaplan–Meier method was used to estimate RFS and OS. The Fisher’s exact test, Wilcoxon rank sum test and log-rank test were used to compare between patients grouped by intervention arm for categorical, continuous and time-to-event results. Confidence intervals were calculated for outcomes of interest. Patients were analysed based on the intervention group to which they were allocated, as per the intention-to-treat (ITT) principle. All tests and confidence intervals were two-sided and a *p*-value of 0.05 or less was considered statistically significant. No interpolation was performed for missing data and exact tests and confidence intervals are presented with no adjustment for multiple testing.

## Results

### Baseline patient, bone health, tumour and treatment characteristics

**Fig. 1:**
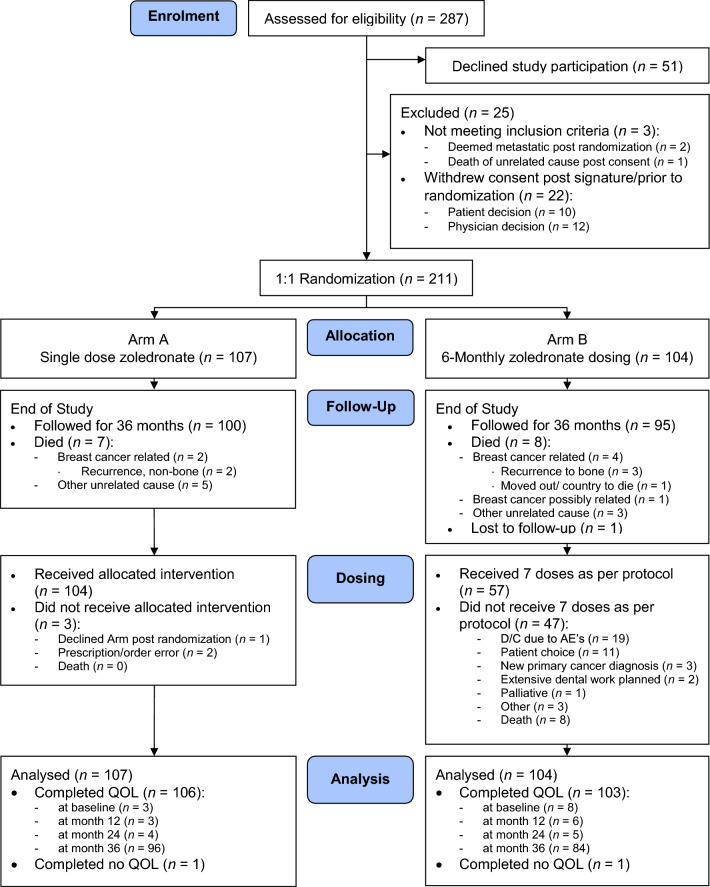
Consort flow diagram

Between November 1, 2018 and April 1, 2020, 287 patients were approached for this study. Of these, 211 were eligible, consented and randomized to either a single infusion (n = 107) or 6-monthly treatments (n = 104) (Supplemental Materials Table S2). A CONSORT diagram is shown in Fig. 1. Baseline patient, bone health, tumour and treatment characteristics of the randomized patients were well balanced and are shown in Table 1 and Supplemental Materials Table S3.

**Table 1:** Baseline patient, bone health, tumour, and treatment characteristics

Variable		All patients	Single infusion arm	6-monthly infusion arm
		211	107	104
Patient characteristics				
Sex (*N *%)	Female	209 (99.1)	107 (100)	102 (1.9)
Age at randomization (Range)	Median	59 (51, 66)	61 (50, 66)	58 (51, 67)
Ethnicity (*N *%)	African	8 (3.8)	7 (6.5)	1 (1.0)
	Asian	17 (8.1)	11 (10.3)	6 (5.8)
	Caucasian	172 (81.5)	83 (77.6)	89 (85.6)
	Native	1 (0.5)	1 (0.9)	0 (0.0)
	Unknown	13 (6.2)	5 (4.7)	8 (7.7)
Menopausal status at cancer diagnosis (*N *%)	Post-	155 (74.2)	77 (72.0)	78 (76.5)
	Peri-	14 (6.7)	7 (6.5)	7 (6.9)
	Pre-	40 (19.1)	23 (21.5)	17 (16.7)
Baseline bone health characteristics				
Osteoporosis (*N *%)	Yes	18 (8.6)	9 (8.4)	9 (8.7)
Osteopenia (*N *%)	Yes	9 (4.3)	2 (1.9)	7 (6.8)
	Don’t know	34 (16.2)	25 (23.4)	9 (8.7)
Bisphosphonate us prior to study entry (*N *%)	Yes	5 (2.4)	1 (0.9)	4 (3.9)
FRAX 10-yr MO fracture score (IQR)	Median	5.7 (3.0, 10.0)	6 (2.8, 9.9)	5.4 (3.3, 10.0)
FRAX 10-yr HF score (IQR)	Median	0.5 (0.2, 1.3)	0.5 (0.1, 1.5)	0.5 (0.2, 1.3)
Tumour characteristics				
TNM stage (*N *%)	IA	55 (26.2)	35 (32.7)	20 (19.4)
	IIA-B	129 (61.4)	55 (51.4)	75 (71.8)
	IIIA-C	26 (12.4)	16 (15.9)	9 (8.7)
ER status (*N *%)	Positive	177 (83.9)	86 (80.4)	91 (87.5)
PR status (*N *%)	Positive	139 (65.9)	69 (64.5)	70 (67.3)
ER and/or PR (*N *%)	Positive	180 (85.3)	87 (81.3)	93 (89.4)
HER2 status (*N *%)	Positive	48 (22.8)	23 (21.5)	25 (24.0)
Triple negative (*N *%)	Yes	26 (12.3)	18 (16.8)	8 (7.7)
Treatment characteristics				
Chemotherapy (*N *%)	Yes	150 (71.1)	79 (73.8)	71 (68.3)
Endocrine therapy (*N *%)	Yes	172 (81.5)	83 (77.6)	89 (85.6)
Endocrine therapy + LHRH analogue (*N *%)	Yes	7 (3.3)	4 (3.7)	3 (2.9)
Trastuzumab (*N *%)	Yes	46 (21.8)	23 (21.5)	23 (22.1)

Median patient age was 59 years (IQR range 51 to 66) with baseline disease stage I (55/210, 26.2%), stage II (129/210, 61.4%) or stage III (26/210, 12.4%). Menopausal status at time of cancer diagnosis was: postmenopausal (155/209, 74.2%), perimenopausal (14/209, 6.7%) and premenopausal who were on ovarian function suppression (40/209, 19.1%). Most patients (165/210, 78.6%) were undergoing regular dental assessments at baseline. A history of mouth pain was reported in 20/209 (9.7%) of patients.

### Quality of life

All components of the EQ-5D-5L remained relatively stable over the 3 years of evaluation, with no statistically significant difference between study arms (Table 2). For example, the median (IQR) “Health Today” scores showed no significant difference between the two study arms at Baseline, Month 12, Month 24 or Month 36. EQ-5D-5L scores were also evaluated before and after each zoledronate infusion and showed no significant changes either before or after each infusion or over time when compared with the single infusion arm (Supplemental Materials Fig. S1).

**Table 2:** QOL (EQ-5D-5L) data over time

Variable	Time	Single infusion	6-Monthly infusion	*p*-value
*N* (%) with moderate or worse pain	Baseline	21/95 (22.1)	19/92 (20.7)	0.86
	Month 12	20/93 (21.5)	22/81 (27.2)	0.48
	Month 24	22/94 (23.4)	26/81 (32.1)	0.24
	Month 36	38/94 (40.4)	29/76 (38.2)	0.87
Median (IQR) health today	Baseline	80 (70, 90)	80 (70, 90)	0.45
	Month 12	85 (75, 90)	80 (73, 92)	0.85
	Month 24	85 (75, 90)	80 (75, 90)	0.62
	Month 36	85 (75, 95)	80 (75, 90)	0.56

### Bisphosphonate-associated toxicities

**Fig. 2:**
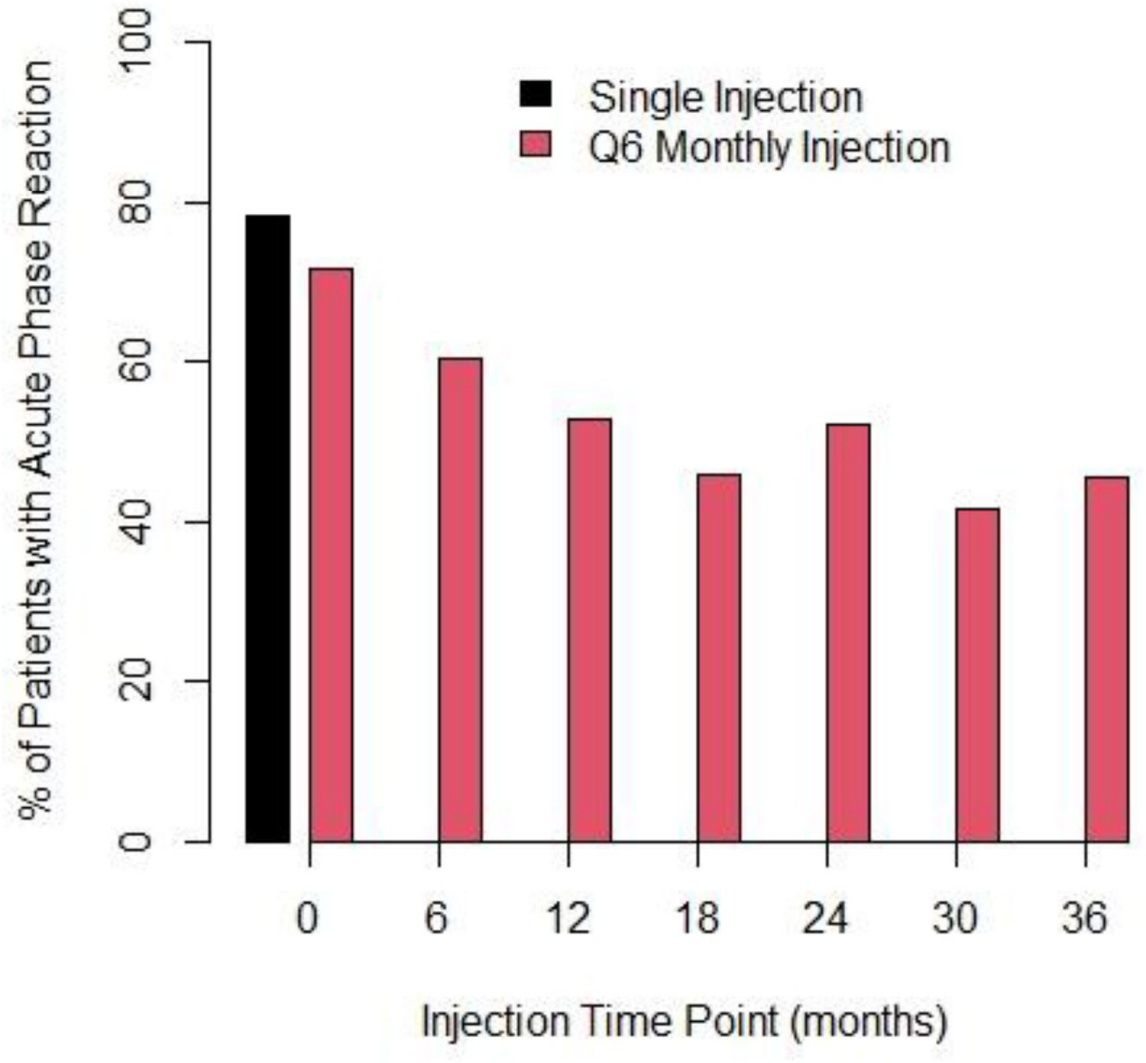
Incidence of acute phase reaction (APRs) following zoledronate infusions

Bisphosphonate-associated toxicities occurring with the first year are shown in Supplemental Materials Table S4. By 3 years of follow up, APRs were the most common toxicity and occurred in 81% (171/211) of all patients after a zoledronate infusion (Table 3). In the single infusion group, 77.6% (83/107) had APRs after their single infusion compared with 84.6% (88/104) in the 6-monthly treatment arm at the three-year assessment (P = 0.005). While the frequency of APRs decreases over the entire 3 years in the 6-monthly arm, they still remained common (Fig. 2).

**Table 3:** 3-Year bisphosphonate-associated toxicity, bone health, and study treatment discontinuation outcomes

Variables	Incidence	All	Single injection	6-Monthly injection	*P*-value
Toxicities within 3 years					
Any bisphosphonate-associated toxicity (*N *%)	Yes	173 (82.0)	83 (77.6)	90 (86.5)	0.11
Acute phase reactions					
Acute phase reactions (*N *%)	Yes	171 (81.0)	83 (77.6)	88 (84.6)	0.005
Consequences of acute phase reaction					
Needed medical attention (*N *%)	Yes	20 (9.5)	9 (8.4)	11 (10.6)	0.64
Oncologist (*N *%)	Yes	10 (4.7)	4 (3.7)	6 (5.8)	0.53
Family physician (*N *%)	Yes	8 (3.8)	4 (3.7)	4 (3.9)	1.00
Attended ER (*N *%)	Yes	4 (1.9)	2 (1.9)	2 (1.9)	1.00
Hospitalisation (*N *%)	Yes	0 (0.0)	0 (0.0)	0 (0.0)	–
Other bisphosphonate-associated toxicities					
Renal (*N *%)	Yes	2 (1.0)	0	2 (1.9)	0.24
Hypocalcemia (*N *%)	Yes	11 (5.2)	1 (0.9)	10 (9.6)	0.22
ONJ (*N *%)	Yes	0 (0.0)	0 (0.0)	0	–
Atrial fibrillation (*N *%)	Yes	0 (0.0)	0 (0.0)	0	–
Atypical fractures (*N *%)	Yes	0 (0.0)	0 (0.0)	0	–
Bone health reported outcomes					
Mouth pain (*N *%)	Yes	36 (17.1)	20 (18.7)	16 (15.4)	0.59
Mouth sores (*N *%)	Yes	5 (2.4)	4 (3.7)	1 (1.0)	0.37
Fragility fractures (*N *%)	Yes	5 (2.4)	4 (3.7)	1 (1.0)	0.37
Patient getting shorter (*N *%)	Yes	31 (14.7)	18 (16.8)	13 (12.5)	0.44
Osteoporosis (*N *%)	Yes	6 (2.8)	5 (4.7)	1 (1.0)	0.21
Discontinued study treatment (*N *%)	Yes	38 (18.0)	4 (3.7)	34 (32.7)	< 0.001
Reasons for discontinuation	AE, pain, unwell		0	16	N/A
	Dental work		0	2	
	PD, palliative		2	1	
	New primary		0	2	
	Patient choice		0	11	
	No reason given		2	2

There were additional consequences of APRs with 9.5% (20/211) of patients requiring additional medical attention. This included: additional visits to either their oncologist (10/211, 4.7%), their family physician (8/211, 3.8%) or emergency room (4/211, 1.9%). The APRs symptoms were the most commonly reported adverse effects and main cause for discontinuation, being responsible for 19 of 38 (49%) discontinuations in the 6-monthly infusions group (Table 3). Other bisphosphonate associated toxicities were uncommon in both arms (Table 3). Despite 36/211 (17.1%) of patients reporting mouth pain and 5/211 (2.4%) reporting mouth sores there were no confirmed cases of ONJ. Hypocalcaemia was reported in 5.2% of patients (Table 3) and was detected as an asymptomatic laboratory finding.

### Bone health reported outcomes

BMD results were available for 28 patients. The incidence of new diagnoses of osteoporosis and fragility fractures at 3 years was 2.8% (6/211) and 2.4% (5/211) respectively in the single infusion and 6-monthly treatment arms respectively (Table 3). There was no difference between the study arms.

### Recurrence-free survival, bone metastasis-free survival and overall survival at 3 years

**Fig. 3:**
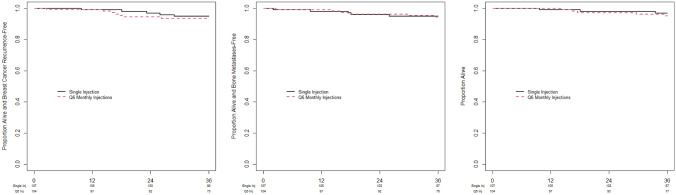
3 Year recurrence-free survival (RFS), bone metastasis-free survival (BMFS) and overall survival (OS) rates

After 3 years of follow-up, there were no statistically significant differences in: RFS (93.4% vs 94.8%), BMFS (94.2% vs 94.9%) and OS (95.1% vs 96.8%) in the single-infusion arm vs the 6-monthly infusion arm (Table 4; Fig. 3). Fifteen patients died during the first 3 years of follow up, 7 in the single-infusion group and 8 in the 6-monthly arm. Mortality reasons were: new different primary cancer (n = 4, bladder, ovary, head and neck); new contralateral cancer (n = 1); recurrent breast cancer (n = 5); non-cancer related (n = 4) causes including cirrhosis, renal failure, COVID, and heart disease). One patient moved out of the country for end-of-life care, but information about their death could not be confirmed.

**Table 4:** 3-Year recurrence-free survival (RFS), bone metastasis-free survival (BMFS) and overall

Variable	All patients	Single infusion arm	6-Monthly infusion arm	*P-*value
Bone metastasis-free survival				
*N* (%) Events	17 (8.1)	8 (7.5)	9 (8.6)	0.96
1-year (95% CI)	98.5 (96, 100)	99.1 (93, 100)	98.0 (92, 99)	
2-year (95% CI)	96.1 (92, 98)	96.2 (90, 99)	95.9(90, 98)	
3-year (95% CI)	95.1 (91, 97)	94.2 (88, 97)	94.9 (88, 98)	
Recurrence-free survival				
*N* (%) Events	18 (8.5)	9 (8.4)	9 (8.6)	0.78
1-year (95% CI)	99.0 (96, 100)	99.1 (93, 100)	99.0 (93, 100)	
2-year (95% CI)	95.6 (92, 98)	94.3 (88, 97)	96.9 (91, 99)	
3-year (95% CI)	94.1 (89, 97)	93.4 (87, 97)	94.8 (88, 98)	
Overall survival				
*N* (%) Deaths	15 (7.1)	7 (6.5)	8 (7.7)	0.87
1-year (95% CI)	99.5 (97, 100)	100	98.9 (93, 100)	
2-year (95% CI)	97.5 (94, 99)	97.1 (91, 100)	97.9 (92, 99)	
3-year (95% CI)	95.9 (92, 98)	95.1 (89, 98)	96.8 (90, 99)

### Study discontinuation

Four patients (3.7%) in the single-infusion arm and 34 (32.7%) in the 6-monthly arm discontinued zoledronate before completion of all treatments in their allocated arm (Table 3). In the 6-monthly arm, 16/34 patients (47%) discontinued because of toxicity (reported “pain” and “unwellness”). Other reasons for stopping zoledronate before completion of all 7 treatments in the 6-monthly arm were: patient choice (11 patients), recurrence (3 patients) and need for dental work (2 patients).

## Discussion

With the results of randomised trials and meta-analyses showing enhanced distant recurrence, bone recurrence and breast cancer mortality rates, adjuvant bisphosphonates are widely recommended for the treatment of postmenopausal patients with breast cancer [1–6]. However, while 6-monthly adjuvant zoledronate is the most commonly used schedule in clinical practice, important questions related to its use remain. Surveys suggest that barriers to wider adoption of adjuvant bisphosphonate use include concerns around; toxicity, funding, efficacy, identification of optimal agent and the requirement for repeat visits for intravenous administration [7, 8, 33]. Indeed, with respect to patient convenience each zoledronate infusion requires regular dental assessments [34], blood work [35], time for drug infusions [7, 8] as well as needs resulting from complications from APRs [36–38]. Identifying the optimal schedule of treatment could enhance care for many patients [1].

The current study identified toxicity rates consistent with the literature. While these agents are usually well tolerated, the incidence of APRs in this study was 87%, higher than the 30% to 57% rates reported in other studies [3, 5, 11–13, 15]. This likely reflects that in the current study, patients receiving zoledronate were specifically telephoned a few days after their zoledronate infusion and were asked questions directly related to this toxicity. This higher incidence likely reflects the fact that on review of the literature few studies have specifically investigated APRs in the adjuvant setting. Indeed, it would be extremely challenging in the setting of a large adjuvant trial to telephone patients before and after each infusion of zoledronate to ask specific questions related to APRs. In reality, most adjuvant trials used the Common Terminology Criteria for Adverse Events criteria for variables such as, “pain” as a surrogate for APRs [3, 5, 11–13, 15]. These measures are likely too insensitive to reflect what is commonly experienced by patients and observed by healthcare providers as APRs. For this reason, the current study used the more sensitive tool developed and valuated by Reid et al. [29]. Similar to the findings of Reid et al. albeit it in the osteoporosis setting [29], if APRs are accurately measured after each zoledronate infusion, their incidence decreases over the three years of treatment but they are still relatively common. This is different from clinical practice where we usually reassure patients having APRs that their incidence and severity decreases with time. This too likely reflects the more sensitive measurement of APRs performed in the current study as well as the effects of other treatments the patients are on, such as aromatase inhibitors. Indeed, the paucity of patients seeking additional medical advice as a consequence of APRs would suggest that despite their incidence, they rarely impacted patient wellbeing. The lack of change observed with the EQ-5D-5L Quality of Life measure likely reflects that it may not be a sensitive as a tool for measuring APRs or that completion of this questionnaire was performed once the APR was over and therefore not captured. However, despite this APRs were the main reason that patients discontinued treatment before completion of all 7 planned cycles.

A single injection of zoledronate could be assumed to have advantages over the 6-monthly treatment for both the patient (enhanced adherence to treatment, reduce visits to the cancer centre for treatment, fewer adverse events) and the healthcare system (significantly reduced costs and resource utilization). While the lack of difference between the study are with respect to the occurrence of fragility fractures in not surprising at the 3 year analysis point, longer term follow up continues. These results will be interesting as the Oxford meta-analysis of individual patient data had available information on fractures from 13,341 (71%) of 18,766 women. Among them, 422 (6.3%) of 6649 bisphosphonate-allocated patients had a fracture, compared with 487 (7.3%) of 6692 control patients (RR 0.85, 95% CI 0.75–0.97; 2p = 0.02), and the 5 year fracture risk was reduced from 6.3 to 5.1% [6]. Reduction in the risk of clinical fractures in postmenopausal women with breast cancer receiving aromatase inhibitors and adjuvant denosumab 60 mg twice per year has also been reported [39]. Similarly, while accepting the RFS, BMFS and OS data are early and the study is not powered for non-inferiority, excellent survival was observed in both arms. These findings are clearly reassuring; however, longer term follow up is ongoing.

Our trial has limitations. The unblinded nature of the study means that both patients and researchers contacting the patients were aware of the treatment allocation and whether they were contacting before or after an infusion thus resulting in risk for bias. While the only way round this would be for the study to be blind we feel we reduced this bias by having a specific telephone script for measuring APRs and by specifically asking patients, “Within 3 days of your zoledronate dose, did you feel….”. However, the tool has limitations in that it is the occurrence of any relevant symptom that leads to an APRs being classified as having happened. The tool does not measure the severity or duration of the symptom nor whether it is related to other treatments the patient is receiving. As mentioned, our sample size was not powered to determine iDFS, BMFS or OS differences and therefore these results need to be interpreted with caution. However, although the number of events which cause RFS, BMFS or OS is very small, and there is almost no perceptible difference between arms, these results emphasise the small number of events and high survival of these patients, regardless of treatment. While we cannot establish non-inferiority, and do not have the power to test for non-inferiority, it is evident that it would take a huge number of patients/events to evaluate this in clinical trials. The study was performed in multiple centres in one country and was affected by the COVID-19 pandemic, during which clinic visits became increasingly virtual and many patients chose to delay their zoledronate in an effort to reduce hospital visits. In addition, in keeping with broader clinical practice some patients (2.4%) had received bisphosphonates for other reasons such as osteoporosis. These numbers were small and well balanced between the study arms and therefore unlikely to impact on the study results,

Future studies are required to address the role of denosumab and also explore the barriers to bisphosphonate use from both patients [8] and healthcare provider perspectives [1, 3, 4, 7, 9]. Studies will also need to explore risk factors for APRs and potential strategies to mitigate them. Finally, a novel placebo-controlled trial evaluating the benefit of adjuvant zoledronate while addressing the questions above is clearly justified given the uncertainty around the efficacy of these agents in the modern era of more effective anti-cancer therapy [40], and the paucity of data supporting 6-monthly zoledronate in the adjuvant setting [3, 11]. Indeed, a recent analysis of the ABCSG 12 trial, albeit limited by the relatively small number of patients not receiving the planned number of zoledronate infusion, had results showing that the number of zoledronate infusions actually administered did not affect the treatment efficacy [41].

## Conclusions

The optimal dosing interval and duration for adjuvant zoledronate remains unknown. While this study is limited by its small sample size, it provides a practical analysis of bisphosphonate-toxicity in a contemporary world and aids oncologists in the management and prediction of adverse effects. While acknowledging that this study was now powered for non-inferiority it provides an interesting signal around the efficacy of a single infusion of zoledronate in the adjuvant treatment of breast cancer.

## Supplementary Information

Below is the link to the electronic supplementary material.

Supplementary file1 (DOCX 96 KB)

## Data Availability

The de-identified study dataset is available upon request to the Principal Investigator (mclemons@toh.ca) with permission from the Ontario Cancer Research Ethics Board  (OCREB).
