# Supplementary material for: A randomised trial comparing 6-monthly adjuvant zoledronate with a single one-time dose in patients with early breast cancer
Source: Breast Cancer Res Treat. 2024 Jul 31;208(3):523–33. doi: 10.1007/s10549-024-07443-2 (PMC11522049; doi:10.1007/s10549-024-07443-2)
Supplement: Supplementary file 1 — Supplementary file1 (DOCX 96 KB) [file 10549_2024_7443_MOESM1_ESM.docx]

**Supplemental Materials Figure S1. Quality of life (EQ-5D-5L) before and after each zoledronate infusion.**


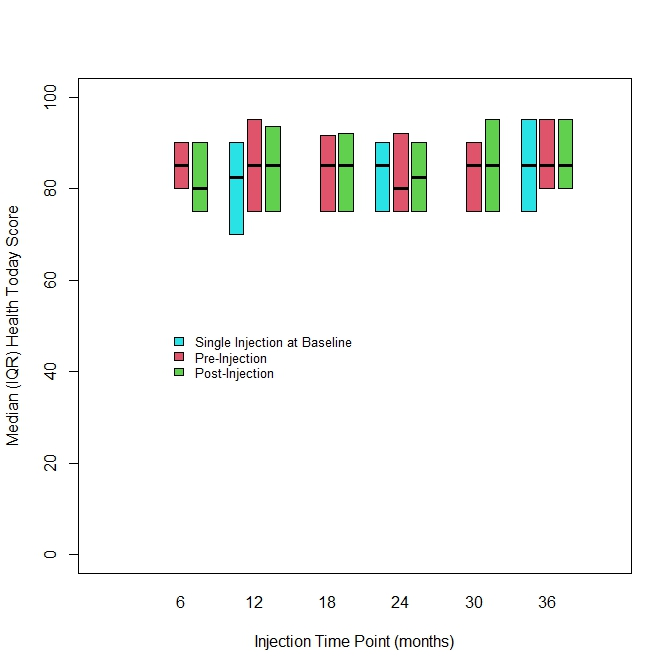


**Supplemental Materials Table S1. Randomised trials with adjuvant 6-monthly zoledronate.** APR = Acute phase reactions; BMD = Bone mineral density; DFS = Disease-free survival; EBC = Early breast cancer; OS = Overall survival.

| **Study** | **Eligibility** | **Randomization Arms** | **Primary Endpoint** | **BMD** | **Toxicity** | **DFS and OS** |
| --- | --- | --- | --- | --- | --- | --- |
| **Zoledronate Versus No Zoledronate** | | | | | | |
| **ABCSG-12**  **Gnant et al. (3)**  **Gnant et al. (11)** | Premenopausal women with endocrine-receptor-positive EBC (stage I–II) breast cancer receiving goserelin (3·6 mg every 28 days) (*n*=1803) | Tamoxifen (*n*=451) Anastrozole (*n*=453)  Tamoxifen plus zoledronate 4 mg every 6 months for 3 years (*n*=449) Anastrozole plus zoledronate (*n*=450) | Primary endpoint: DFS  Secondary endpoints: Recurrence free survival, overall survival and change in BMD | At 5 years:  No zoledronate arm:  -6.3%  Zoledronate arm: 4% | APR: 30% of patients receiving zoledronate  Renal: Not reported  ONJ: Not reported  Hypocalcaemia: Not reported | At 76 months (3):  Zoledronate arm:  27% improvement in DFS  41% improvement in OS  At 7.8 years (11):  DFS 88% versus 85%, (*P* = 0.203)  No OS benefit |
| **Upfront Zoledronate Versus Delayed Zoledronate** | | | | | | |
| **N03CC (Alliance)**  **Wagner-Johnston et al. (13)** | Postmenopausal women with EBC who have completed tamoxifen and starting letrozole (*n*=551) | Upfront zoledronate (4 mg IV every 6 months) (*n*=274)  Delayed zoledronate started if post-baseline BMD T-score < -2.0 or fracture (*n*=277)  15% of 277 received zoledronate | Primary endpoint: change in BMD  Secondary: Incidence of Osteoporosis and Fractures | At 12 months:  Upfront: gain of 3.6%  Delayed: loss of 1.6% | APR:  Upfront- 57%  Delayed- 33%  Creatinine elevation: Upfront- 4%  Delayed- 1%  ONJ:  Upfront- 0.2%  Delayed- 0%  Hypocalcaemia: not reported | Not reported |
| **Z-FAST**  **Brufsky et al. (12)** | Postmenopausal women with receptor–positive EBC receiving adjuvant letrozole were randomized to receive up-front or delayed-start zoledronic acid. Delayed-start ZA was administered | Upfront zoledronate (4 mg IV every 6 months for 5 years) (*n*=301)  Delayed zoledronate started if lumbar spine (LS) or total hip (TH) T score fell below −2.0 or a nontraumatic fracture occurred (*n*=301)  16.3% of 301 received zoledronate | Primary endpoint: Change in BMD  Secondary endpoints: Non traumatic fractures and DFS | At 5 years:  Upfront: loss of 2 %  Delayed: loss of 25.7% | APR:  Upfront: 47%  Delayed: 45.3%  Renal impairment: Upfront: 2%  Delayed: 1.3%  ONJ: 0.16% | At 5 years:  DFS  Upfront: 95.7%  Delayed: 93%  OS  Upfront: 97.7%  Delayed: 98.7% |
| **ZO-FAST**  **Eidtmann et al. (5)** | Postmenopausal women with receptor–positive EBC receiving adjuvant letrozole for 5 years | Upfront zoledronate (4 mg IV every 6 months for 5 years) (*n*=524)  Delayed zoledronate started if T-score fell below -2.0, nontraumatic clinical fracture, or if an asymptomatic fracture detected by spinal X-ray at the 36- month assessment. (*n*=536)  27% of 536 received zoledronate | Primary endpoint: change in BMS after 12 months.  Secondary endpoints: change in total BMD at each assessment, 3-year fracture incidence and DFS | At 4 years:  Upfront: +4.3  Delayed: -5.4% | APR:  Upfront: 46.6%  Delayed: 45.6%  Renal: Not reported  ONJ: 0.1%  Hypocalcaemia: Not reported | At 4 years:  DFS  Upfront: 99%  Delayed: 98%  OS  Upfront: 95%  Delayed: - 93.9% |
| **KCSG-BR06-01**  **Kim et al. (14)** | Premenopausal women aged >40 years with EBC receiving chemotherapy | Upfront zoledronate (4 mg IV, starting on the day of first adjuvant chemotherapy, every 6 months for 12 months (*n*=57)  Delayed zoledronate started if; atraumatic fracture or 6 month follow-up BMD T-score -2.5 standard deviations (SDs) at either the LS or total hip (*n*=59)  Does not report how many received zoledronate | Primary endpoint: change in BMD at 12 months  Secondary endpoints: changes in bone turnover markers and adverse effects of zoledronate in combination with chemotherapy | Upfront: -1.1%  Delayed: -7.5% | APR: Numbers not reported  Renal: Not reported  ONJ: 0  Hypocalcaemia: Not reported | Not reported |
| **Takahashi et al. (15)** | Postmenopausal women with receptor–positive EBC receiving adjuvant letrozole for 5 years | Upfront zoledronate (4 mg or an adjusted dose based on renal function IV every 6 months for 5 years) (*n*=97)  Delayed zoledronate started if post baseline LS (L2–L4) BMD decreases to YAM -2.0SD or less, or a nontraumatic clinical fracture occurred. (*n*=97)  Does not report how many received zoledronate | Primary endpoint: change in BMD at 12 months  Secondary endpoints: change in bone turnover markers, fracture at 3 years and DFS | Upfront: of the pts who already had bone loss at baseline, 22.2% recovered BMD to normal and 0% developed osteopenia  Delayed: 5.1% of pts who had bone loss at baseline recovered BMD to normal and 5% developed osteopenia. | APR  Upfront: 33.2%  Delayed: 11.4%  Renal: Not reported  ONJ:0  Hypocalcaemia: Not reported | Not reported |

**Supplemental Materials Table S2. Number of infusions of zoledronate received.**

| **Randomized to single dose** | **107** | # receiving correct allocation = 104 |
| --- | --- | --- |
|  |  | # received more than single = 3   - Received > 1 in error (2) -verbal orders by MO - Received >1 pt choice (1)-switch to SOC   **Total = 107** |
| **Randomized to SOC** | **104** | # receiving correct allocation = 57 |
|  |  | # receiving less than allocation = 47 |
|  |  | **Total = 104** |
| **TOTAL PATIENTS** | **211** | **211** |
|  |  |  |
| **Doses of zoledronate received:** |  |  |
| 1 | 119 |  |
| 2 | 10 |  |
| 3 | 2 |  |
| 4 | 6 |  |
| 5 | 7 |  |
| 6 | 10 |  |
| 7 | 57 |  |
| **TOTAL PATIENTS** | **211** |  |

**Supplemental Materials Table S3. Variables incorporated into FRAX score and Quality of life (EQ-5D-5L) at baseline.**

| **Variable** |  | ***N*** | **All Patients** | **Single Zoledronate Infusion Arm** | **Q6-Monthly Infusion Arm** |
| --- | --- | --- | --- | --- | --- |
| ***N*** |  |  | 211 | 107 | 104 |
| **Variables Incorporated Into FRAX Score** | | | | | |
| Confirmed RA | *N* (%) Yes | 211 | 6 (2.8) | 4 (3.7) | 2 (1.9) |
| Previous fracture | *N* (%) Yes | 209 | 7 (3.4) | 4 (3.8) | 3 (2.9) |
| Hip fracture | *N* (%) Yes | 209 | 27 (12.9) | 11 (10.4) | 16 (15.5) |
| Current smoker | *N* (%) Yes | 209 | 9 (4.3) | 3 (2.8) | 6 (5.8) |
| Alcohol | *N* (%) Yes | 210 | 7 (3.3) | 4 (3.7) | 3 (2.9) |
| Secondary osteoporosis | *N* (%) Yes | 211 | 1 (0.5) | 0 | 1 (1.0) |
| Corticosteroids | *N* (%) Yes | 211 | 1 (0.5) | 0 | 1 (1.0) |
| Previous oral steroids | *N* (%) Yes | 211 | 1 (0.5) | 0 | 1 (1.0) |
| Type I diabetes | *N* (%) Yes | 211 | 1 (0.5) | 1 (0.9) | 0 |
| Osteogenesis imperfecta | *N* (%) Yes | 211 | 0 (0) | 0 | 0 |
| Hyperthyroidism | *N* (%) Yes | 211 | 1 (0.5) | 0 | 1 (1.0) |
| Premature menopause | *N* (%) Yes | 211 | 6 (2.8) | 4 (3.7) | 2 (1.9) |
| Chronic malnutrition | *N* (%) Yes | 211 | 1 (0.5) | 1 (0.9) | 0 |
| Chronic liver disease | *N* (%) Yes | 211 | 3 (1.4) | 2 (1.9) | 1 (1.0) |
| **Quality of Life at Baseline** | | | | | |
| **Mobility** | *N* (%) None  Slight  Moderate | 194 | 139 (71.7)  43 (22.2)  12 (6.2) | 69 (70.4)  24 (24.5)  5 (5.1) | 70 (72.9)  19 (19.8)  7 (7.3) |
| **Self-care** | *N* (%) None  Slight  Moderate  Severe | 194 | 179 (92.3)  11 (5.7)  2 (1.0)  2 (1.0) | 91 (92.9)  6 (6.2)  1 (1.0)  0 (0) | 88 (91.7)  5 (5.2)  1 (1.0)  2 (2.1) |
| **Usual activities** | *N* (%) None  Slight  Moderate  Severe  Extreme | 194 | 100 (51.6)  13 (6.7)  13 (6.7)  1 (0.5)  1 (0.5) | 52 (53.1)  37 (37.8)  8 (8.2)  1 (1.0)  0 (0) | 48 (50.0)  42 (43.8)  5 (5.2)  0 (0)  1 (1.0) |
| **Pain** | *N* (%) None  Slight  Moderate  Severe  Extreme | 194 | 74 (38.1)  90 (46.4)  27 (13.9)  2 (1.0)  1 (0.5) | 36 (36.7)  47 (48.0)  13 (13.3)  2 (2.0)  0 (0) | 38 (39.6)  43 (44.8)  14 (14.6)  0 (0)  1 (1.0) |
| **Anxiety/Depression** | *N* (%) None  Slight  Moderate  Severe  Extreme | 194 | 104 (53.6)  72 (37.1)  14 (7.2)  2 (1.0)  2 (1.0) | 60 (61.2)  32 (32.7)  6 (6.1)  0 (0)  0 (0) | 44 (45.8)  40 (41.7)  8 (8.3)  2 (2.1)  2 (2.1) |
| **Health today** | Median (IQR) | 194 | 80 (70, 90) | 85 (70, 90) | 80 (70, 90) |

**Supplemental Materials Table S4. Bisphosphonate-associated toxicity, breast cancer recurrence and study treatment discontinuation outcomes within year 1 after randomisation.**

| **Variable** |  | **All Patients** | **Single Infusion Arm** | **Q6-Monthly Infusion Arm** | **p-value** |
| --- | --- | --- | --- | --- | --- |
| **Toxicities Within 1 Year** | | | | | |
| **Any bisphosphonate-associated toxicity** | *N* (%) Yes | 163 (77.3) | 82 (76.6) | 81 (77.9) | 0.87 |
| **Acute Phase Reactions** | | | | | |
| **Acute phase reaction** | *N* (%) Yes | 162 (76.8) | 82 (76.6) | 80 (76.9) | 1.00 |
| **Consequence of Acute Phase Reaction** | | | | | |
| **Needed medical att.**  **Oncologist**  **Family physician**  **Attended ER**  **Required hospitalisation** | *N* (%) Yes | 16 (7.6)  6 (2.8)  8 (3.8)  4 (1.9)  0 (0) | 9 (8.4)  4 (3.7)  4 (3.7)  2 (1.9)  0 | 7 (6.7)  2 (1.9)  4 (3.9)  2 (1.9)  0 | 0.80  0.68  1.00  1.00  - |
| **Other Bisphosphonate-Associated Toxicities** | | | | | |
| **Renal**  **Femur**  **Hypocalcemia**  **Atrial Fibrillation** | *N* (%) Yes | 1 (0.5)  0 (0)  5 (2.4)  0 (0) | 0  0  1 (0.9)  0 | 1 (1.0)  0  4 (3.9)  0 | 0.49  -  0.21  - |
| **ONJ**  **Mouth Pain**  **Mouth Sores**  **Fractures**  **Shorter**  **Osteoporosis** | *N* (%) Yes | 0 (0)  15 (7.1)  1 (0.5)  1 (0.5)  8 (3.8)  0 (0) | 0  10 (9.4)  1 (0.9)  1 (0.9)  5 (4.7)  0 | 0  5 (4.8)  0  0  3 (2.9)  0 | -  0.28  1.00  1.00  0.72  - |
| **BR Ca Recurrence**  **Bone Mets** | *N* (%) Yes | 1 (0.5)  2 (1.0) | 1 (0.9)  1 (0.9) | 0  1 (0.9) | 1.00  1.00 |
| **Discontinued Study Treatment** | *N* (%) Yes | 16 (7.6) | 3 (2.8) | 13 (12.5) | 0.009 |
